# Nanomaterials Applied to Fuel Cells and Catalysts

**DOI:** 10.3390/nano15050347

**Published:** 2025-02-23

**Authors:** András Tompos

**Affiliations:** Institute of Materials and Environmental Chemistry, HUN-REN Research Centre for Natural Sciences, Magyar Tudósok Körútja 2, H-1117 Budapest, Hungary; tompos.andras@ttk.hu

## 1. Introduction

Hydrogen and fuel cell technologies are accepted by consensus as being part of the future energy system, especially in hard-to-abate segments where electrification is not an efficient solution. In transport sector, a fuel cell electric powertrain is an excellent choice where long ranges and/or high payloads are required. In stationary power generation (combined heat and power generation, CHP), fuel cells have a higher electricity generation efficiency than most other technologies such as gas turbines and engines. Moreover, fuel cells can also be used for primary power generation, e.g., for data centers and corporate campuses, and for distributed power generation that eliminates losses from the electrical grid. Reversible fuel cells have great potential in coupling energy sectors at gas and electricity grid nodes. In this case, hydrogen, hydrogen–methane blends, biogas, syngas, and ammonia can be used for the power supply, which can also increase the round-trip efficiency of fuel cells, assuming the fuel flexibility of the chosen fuel cell technology.

However, before mass market penetration, further developments are needed to increase lifetime, fuel flexibility, reduce costs, and increase efficiency in order to be competitive with conventional technologies. It is generally accepted that both research and innovation breakthroughs are needed.

Although fuel cells have relatively few key components, such as catalysts, membrane electrode assemblies, bipolar plates, and gas diffusion layers, the material science behind the development of these components is quite complex. The development of new disruptive technologies based on material science is necessary.

For proton exchange membrane fuel cells (PEMFCs), the produced membrane electrode assemblies (MEAs) should demonstrate high durability and power density, and low platinum group metal (PGM) loading. The life time should exceed 30,000 h in transport applications (aviation, heavy goods vehicles, rail, marine, and/or passenger vehicles), achieve a power density of 1.2 W/cm^2^, and a PGM load below 0.3 g/kW. For large-scale production of MEAs, the cost target for road and rail applications is <50 EUR/kW in 2030 [1].

New fuel cell catalysts, free of critical raw materials or unsustainable or environmentally unacceptable constituents, need to be developed, characterized, and validated in fuel cells without compromising their performance and durability. MXenes, one of the prominent representatives of 2D materials composed of transition metal carbides, nitrides, and carbonitrides, have attracted interest as potential electrocatalysts [2]. MOFs are generally not used as electrocatalysts due to their poor electrical conductivity. However, carbon-based MOF derivatives are widely used materials in electrocatalysis. The tunable molecular structure, high conductivity, and environmental compatibility of carbon-based MOF materials allow, in addition to good electrical conductivity, for fine-tuning the catalytic activity by combining with other materials that shape the electrocatalytic activity through electronic and geometric effects [3]. Organic small molecules (OSMs) with well-defined structures can be components of fuel cell cathode catalysts [4]. Fe-N-C electrocatalysts have emerged as viable alternatives to platinum [5]. The carbonaceous materials with large surface area are doped with iron and nitrogen. Atomically dispersed Fe-N4 active sites are hypothesized to be the most active ORR sites in acidic media [6,7,8]. The development of single-atom catalysts (SACs) not only reduces the need for critical raw materials, but also holds the promise of developing a synergy between the central active atom and its coordination environment, thereby achieving better catalytic activity [9].

Understanding and mitigating the degradation mechanisms is of utmost importance but, at the same time, the performance of new electrocatalysts must be improved, reducing their dependence on critical raw materials (CRM). Advanced operando techniques have to be developed in order to follow aging mechanisms under real-world conditions (i.e., working temperature, dynamic load, pressure) and in the presence of contaminants (e.g., from fuel and air). To overcome time-consuming durability tests, so-called accelerated durability tests (ADTs) are of urgent need [10]. Additionally, there is a growing need to develop robust degradation models that can accurately predict the durability of fuel cells during operation [11].

In this Special Issue, entitled “Fuel Cells and Catalysts”, new solutions are offered to reduce costs, increase lifetimes, and enable more efficient operation of the cells.

## 2. An Overview of Published Articles

Santoveña-Uribe et al. (contribution 1) continued to develop fuel cell components by optimizing nanoparticle characteristics, particularly to improve efficiency and reduce the dependence on critical raw materials. The relationship between the mean diameter of palladium (Pd) nanoparticles and their surface energy was investigated, specifically in the context of alkaline ethanol electro-oxidation for fuel cell applications. The surface energy of the Pd/C nanocatalysts showed a linear correlation with the particle size, while remaining independent of the bulk concentration of ethanol. The optimal average diameter of Pd was found to be about 28 nm for enhanced electrocatalytic activity, suggesting that catalytic efficiency is size-dependent. It was pointed out that the competitive adsorption of the ethanol molecule and oxidation intermediates is crucial for the overall electrooxidation mechanism.

The aim of the work of Krasnova et al. (contribution 2) was to comprehensively study the resistance of electrodes based on Graphene/Nafion composites to different types of stressors: electrochemical, chemical, and thermal. Graphene has been shown to inhibit the degradation of Nafion when exposed to heat. Nafion, on the other hand, causes graphene to degrade when exposed to higher temperatures. During electrochemical and chemical exposure, in the case of the carbon-rich composites, the decisive change was carbon loss due to oxidation of the carbon material. In the case of low-carbon composites, the dominant process is the removal of fluorine and sulfur from the Nafion polymer, with their partial replacement with oxygen. In all cases, the F/S ratio is stable. The dispersion of Nafion in the samples affects its chemical stability more than the G/Nafion ratio.

Pt-free electrocatalysts based on the carbonization of glucose were prepared and characterized by Fontanesi et al. (contribution 3). The effects of two hydrothermal synthesis methods on the electrocatalytic performance of the obtained carbonaceous materials in the oxygen reduction reaction (ORR) under alkaline conditions was compared. Despite its rapid processability, the microwave-assisted hydrothermal carbonization (MW-HTC) method results in products with surface chemistry and morphology that are less suitable for ORR than those produced by the conventional hydrothermal carbonization (T-HTC) method. The excellent performance of the catalysts produced by the T-HTC method can be attributed to the particle morphology and the chemical composition of the particle surfaces. The presence of functional groups on the surface of the catalysts produced by the traditional approach significantly enhances the ORR activity by promoting deprotonation reactions in an alkaline environment. It should be emphasized that although the current experimental conditions of MW-HTC thermal treatment did not lead to efficient catalysts, this result can be improved by changing process parameters such as time or temperature.

The work of Massaglia et al. (contribution 4) investigated a novel nanostructured gas diffusion layer (nano-GDL) to improve the performance of air-cathode single-chamber microbial fuel cells (a-SCMFCs). This type of fuel cell is membraneless, and characterized by a single reaction chamber shared between the anode and the cathode. The cathode side needs a multi-functional architecture, i.e., it has to be waterproof, have high oxygen permeability, prevent the leakage of water-based electrolytes, and promote the diffusion of oxygen to the catalytic sites. A carbonized pattern of cellulose nanofibers was proposed by direct laser writing, which played a key role in providing the necessary hydrophilicity to improve water retention near the active catalytic sites, thus avoiding the loss of proton conductivity of the electrolyte by dehydration. For a-SCMFCs, the maximum current density obtained with the new nano-GDLs is twice that of the standard PTFE-based GDL. The energy recovery factor (EF), i.e., the ratio of the output power to the internal volume of the device, was an order of magnitude higher than the value obtained with the standard GDL.

The multi-functionality of a composite electrocatalytic material designed for PEMFCs was again the focus in the work by Silva et al. (contribution 5). Platinum supported on the composites of tin-doped titania and carbon (Pt/Ti_0.8_Sn_0.2_O_2_-C) electrocatalysts were developed. The carbon component contributed to the high electrical conductivity, and served as a hard template with a large surface area; the oxide component stabilized the Pt particles; and the Sn additive provides a co-catalytic function. Accordingly, the goal was the formation of Pt–oxide–C triple junctions. The development of the so-called Strong Metal–Support Interaction (SMSI) was proposed as a means of implementation. The uniform oxide coverage of the carbon backbone facilitated the formation of Pt–oxide–C triple junctions at a high density. The electrocatalytic behavior of the as prepared catalysts was determined by the atomic closeness of Sn to Pt, while even a low-temperature reductive treatment resulted in Sn–Pt alloying. The segregation of tin oxide on the surface of the alloy particles, a characteristic material transport process in Sn–Pt alloys after oxygen exposure, contributed to better stability of the reduced catalysts.

## 3. Conclusions

Among the main goals of the authors of this Special Issue, the creation of more sustainable electrodes with better electrocatalytic efficiency are highlighted. The improved efficiency reduces the required amount of platinum group metal (PGM) to be used, leading to more sustainable solutions. The sustainability problem can also be solved with completely PGM-free catalysts. In both cases’ novel nanostructures, disruptive ideas are required for design of completely new nanomaterials with decreased amounts of critical raw materials.

A specific, although growing, area of fuel cell technology is the microbial fuel cell, where sustainability is achieved by utilizing biological waste and converting it directly into electricity.

The degradation of the components due to various stressors have been investigated, which is of particular importance in terms of the service life of fuel cells. Not only is the operation of fuel cells affects their durability, but the materials used can also influence the chemical, electrochemical, and thermal decomposition processes.

In summary, it can be said that there is room for improvement from a materials science point of view, and technological progress is closely related to the successes achieved by scientists in the field of nanomaterials.
